# Dissecting the control of shoot development in grapevine: genetics and genomics identify potential regulators

**DOI:** 10.1186/s12870-020-2258-0

**Published:** 2020-01-29

**Authors:** Sabine Guillaumie, Stéphane Decroocq, Nathalie Ollat, Serge Delrot, Eric Gomès, Sarah J. Cookson

**Affiliations:** 1 UMR1287 EGFV, Bordeaux Sciences Agro, INRAE, University of Bordeaux, Villenave d’Ornon, France; 2 UMR1332 BFP, INRAE, University of Bordeaux, Villenave d’Ornon, France

**Keywords:** CURLY LEAF, Dwarfed phenotype, F2 population, Quantitative trait loci, Shoot development, *Vitis* interspecific cross

## Abstract

**Background:**

Grapevine is a crop of major economic importance, yet little is known about the regulation of shoot development in grapevine or other perennial fruits crops. Here we combine genetic and genomic tools to identify candidate genes regulating shoot development in *Vitis spp*.

**Results:**

An F2 population from an interspecific cross between *V. vinifera* and *V. riparia* was phenotyped for shoot development traits, and three Quantitative Trait Loci (QTLs) were identified on linkage groups (LGs) 7, 14 and 18. Around 17% of the individuals exhibited a dwarfed phenotype. A transcriptomic study identified four candidate genes that were not expressed in dwarfed individuals and located within the confidence interval of the QTL on LG7. A deletion of 84,482 bp was identified in the genome of dwarfed plants, which included these four not expressed genes. One of these genes was *VviCURLY LEAF* (*VviCLF*), an orthologue of *CLF*, a regulator of shoot development in *Arabidopsis thaliana*.

**Conclusions:**

The phenotype of the dwarfed grapevine plants was similar to that of *clf* mutants of *A. thaliana* and orthologues of the known targets of CLF in *A. thaliana* were differentially expressed in the dwarfed plants. This suggests that CLF, a major developmental regulator in *A. thaliana*, also controls shoot development in grapevine.

## Background

Grapevine (*Vitis spp.*) is one of the most economically important perennial fruit crops cultivated worldwide and the regulation of shoot development is central to fruit yield and berry oenological potential. On a botanical basis, grapevine is a liana with indeterminate growth habits. Commercially grown grapevines produce annual shoots that emerge from a given number of latent buds retained after pruning and shoot development results from the recurrent production and development of phytomers. Like most deciduous woody plants, annual shoot development in grapevine begins with the development of latent buds containing generally six to 10 pre-formed phytomers, followed by the indeterminate production of neo-formed phytomers [1].

The molecular control of shoot development has been well characterised in model species such as *Arabidopsis thaliana*, through the identification and characterisation of mutants and gene networks underlying different shoot developmental phenotypes [2]. In grapevine (*V. vinifera* L.), only a small number of shoot development mutants have been identified in grapevine. One example is the *Vvigai1* dwarf gibberellin insensitive mutant derived from the L1 layer of *V. vinifera* cv. Pinot meunier, which produces extremely short internodes and inflorescences in the place of tendrils along the shoot [3]. Other dwarf grapevines have been identified based on gibberellin insensitivity [4, 5]. These dwarf grapevines are of particular interest for genetic studies in small controlled environments [4].

In addition to gibberellin insensitive mutants described above, dwarf phenotypes have been reported with a high frequency within self-progenies of *V. vinifera* and *V. riparia* hybrids and other interspecific progenies [6, 7]. The underlying mechanisms and the genetic architecture of such abnormalities are not known. However, cultivated, clonally propagated grapevines are known to present a high level of heterozygosity and may carry a heavy load of deleterious recessive alleles; as such they are highly susceptible to inbreeding depression [8]. Plants presenting inbreeding depression symptoms offer the opportunity to understand plant functioning via the identification of the loci and/or the molecular mechanisms potentially involved.

Quantitative Trait Loci (QTL) analysis has been widely used to describe the genetic architecture of phenotypic traits segregating in interspecific and intraspecific crosses. For example, QTLs have been identified for various shoot development related traits in grapevine, particularly those associated with leaf area [5], inflorescence morphology [9], berry development and composition such as weight, colour, sugar or acid contents [5, 10, 11] and phenology [12–15]. To date, the genetic architecture of internode length in grapevine has only been studied on a cross between the Picovine 00C001V0008 (*Vvigai1*/*Vvigai1*, a dwarf vine with a rapid life cycle) and the *V. vinifera* cv. Ugni Blanc fleshless berry mutant [5]. No QTLs repeated over years were found for this trait. Further studies are therefore required to provide new insights into the genetic control of this trait. Screening parents for dwarfism with genetic makers could help to develop efficient breeding programs.

Most QTL studies in grapevine have been performed on F1 populations [16] and only four grapevine genetic maps based on F2 populations have been published [11, 17–19]. Compared to an F1-based mapping strategy, genetic maps developed on F2 populations (with at least 200 progeny) have superior linkage map accuracy and enable the capture of additional meiotic events and recessive allele effects such as those potentially underlying inbreeding depression symptoms [11, 20]. However, previous F2 studies in grapevine have not yet characterised the control of dwarfing traits.

In the present study, we combined QTL mapping with transcriptomics to identify the potential regulators of shoot development in grapevine. The genetic architecture of shoot development was characterised in an F2 population derived from a cross between *V. vinifera* and *V. riparia*. Approximately 17% of this population presented dwarfed phenotypes. The transcripts differentially expressed in the young shoots of plants with dwarfed and normal phenotypes were compared using oligonucleotide microarrays. This led to the identification of a deletion in the genome of *V. vinifera* cv. Cabernet-Sauvignon (CS) which, based on the homology with genes from *A. thaliana*, contains potential shoot development regulators.

## Results

### Genetic linkage map construction

For linkage mapping, a total of 173 simple sequence repeat (SSR) markers were tested with DNA samples from CS, *V. riparia* cv. Gloire de Montpellier (RGM), F1_148 and five individuals of the CS x RGM_F2 population. Among them, 47 new markers were developed for this study (Additional file 1). Four SSR markers had a monomorphic profile (VVMD25, UDV013, VMC3C11, VVBX07), five had a complex or multiloci profile (VVBX13, VVIH02, VVIM72, VVIV70, UDV061) and 18 were not reliably amplified, and were all removed from the analysis.

The remaining 146 polymorphic markers were used to genotype the 337 individuals of the CS x RGM_F2 population and to construct and validate the genetic linkage map by the use of the softwares Carthagene and JoinMap® 3.0 (Additional file 2). Only the VMC2A9 and VVIP17 markers related to multiple loci were kept. When multiple loci were amplified with the same primer pair, the suffix ‘a’ ‘b’ or ‘CS’ ‘RGM’ was added to the marker name (Additional file 2). The average number of individuals genotyped per loci was 336 with a minimum of 325 individuals genotyped.

All the 146 markers were linked and mapped into 19 LG. The total length of the map was 1051.1 centiMorgan (cM) with an average distance of 7.2 cM between markers and 7.68 markers per LG (Additional file 3). Ten gaps larger than 20 cM were identified. The largest gap, between markers VVC34 and VVIP26 on LG 14, was 30.3 cM. Linkage group sizes ranged from 47.4 cM (LG5) to 76.2 cM (LG18) with an average size of 55.3 cM. The marker order was consistent with the order determined from the F1 population CS x RGM1995–1 and from the *V. vinifera* 12X genome sequence.

### QTL detection of shoot development traits

**Fig. 1:**
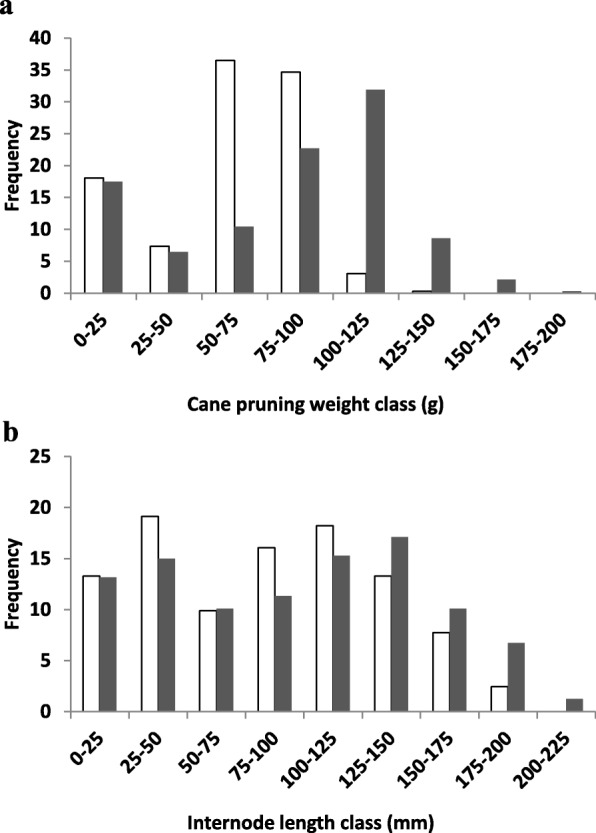
Distribution of the cane pruning weight (CPW) and internode length (IL) traits of the CS x RGM_F2 population. The CPW (**a**) and the IL (**b**) were expressed in g and in mm respectively (white bars 2009, grey bars 2010). Vertical bars indicate standard errors

**Fig. 2:**
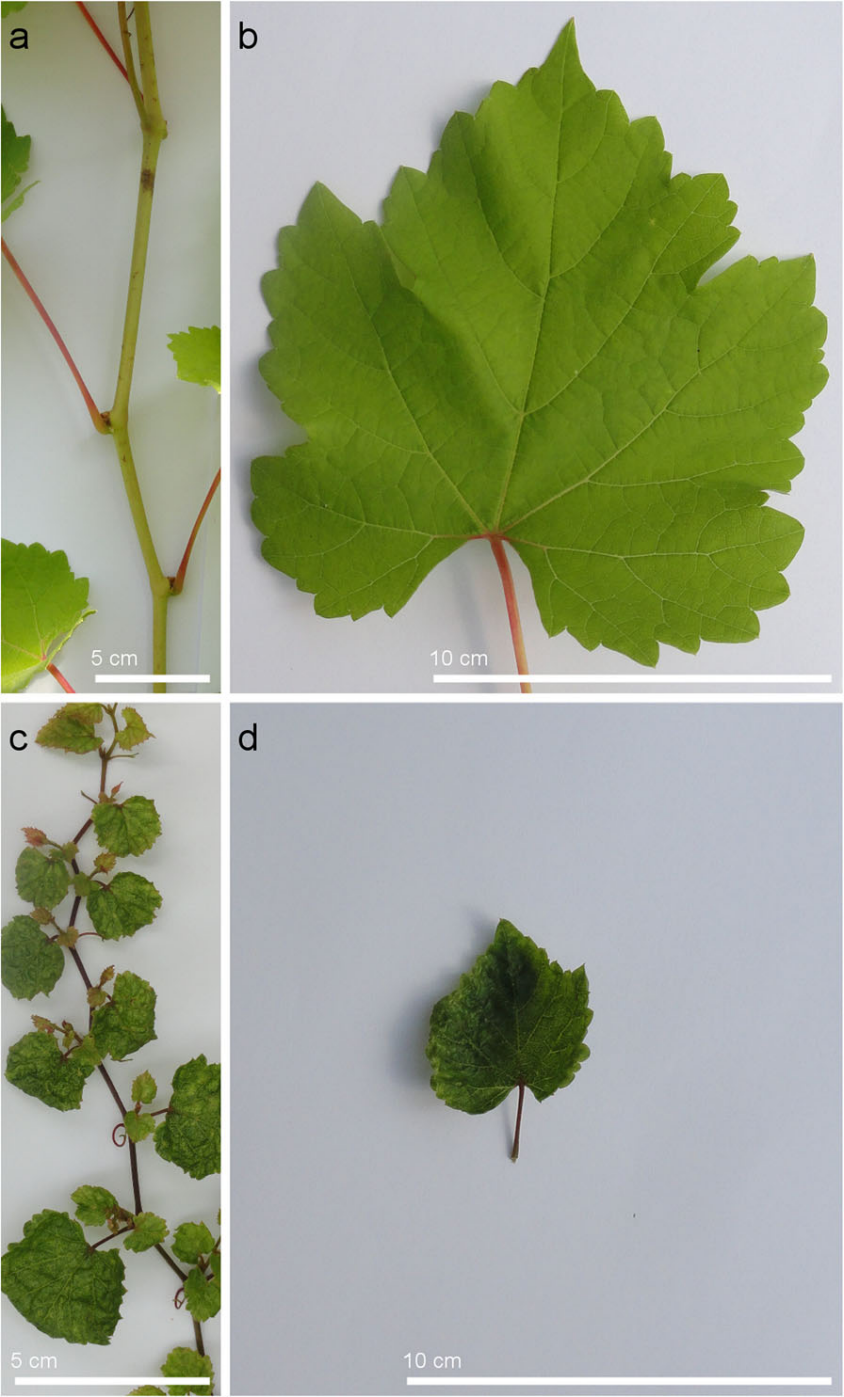
Leaf and internode phenotypes. **a** and **b** “Normal” internode and leaf phenotype of F2_259 genotype. **c** and **d** Dwarfed shoot and leaf of F2_024 plant. Bars: 5 cm (**a**, **c**), 10 cm (**b**, **d**)

To detect QTLs, the winter cane pruning weight (CPW) and internode length (IL) of 326 genotypes of the F2 population were measured. The population showed considerable phenotypic variation for each trait (Fig. 1). They did not display a normal distribution and appeared to be made up of two populations of different sizes, normal and dwarfed. Seventeen percent of the CS X RGM_F2 population was composed of dwarfed plants, defined in this study as having a winter CPW of less than 25 g. In addition, both stem development, and leaf shape and size were affected (Fig. 2; Additional file 4). The dwarfed plants had curled-leaf phenotype (Fig. 2d and Additional file 4e-g) and no flowers.

Pearson correlation coefficients were calculated for each IL and CPW between years and the two traits during a single year (*p* < 0.05). For each correlation tested, a significant positive coefficient was found. Highly significant correlations were found both for each trait between years (r^2^ = 0.80 and 0.86 for winter CPW and IL respectively), and for the different traits in each year (r^2^ = 0.52 and 0.64 for 2009 and 2010 respectively).

**Fig. 3:**
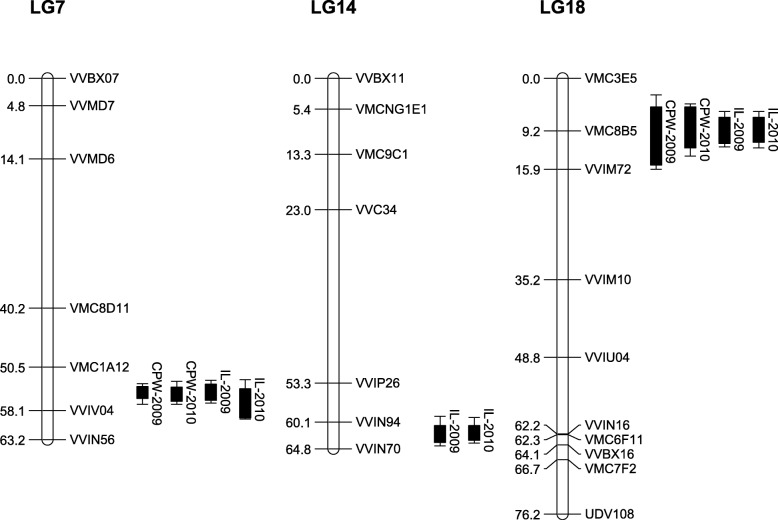
Genomic position of quantitative trait loci (QTLs) detected on the linkage groups of the CS x RGM_F2 map by multiple QTL mapping (MQM). QTLs are represented by boxes extended by lines representing the logarithm of the odds (LOD)-1 and LOD-2 confidence intervals. Linkage groups are named according to international consensus map. CPW, cane pruning weight; LG, linkage group; IL, internode length. Distances are in cM Kosambi

The non-parametric Kruskal–Wallis test suggested the existence of QTLs for CPW and IL on LG7, LG14 and LG18 (Table 1). Then, the Multiple QTL Mapping (MQM) analysis identified 10 significant QTLs of shoot development traits with a significant LOD score > 3.0. Four QTLs were located on LG7 and LG18 for CPW and six were located on LG7, LG14 and LG18 for IL (Table 1; Fig. 3). QTLs detected on LG7 and LG18 for CPW co-located with QTLs for IL detected on same LGs.

**Table 1:** Parameters associated with quantitative trait loci (QTLs) detected by multiple QTL mapping (MQM) for vegetative variables measured on the CSxRGM_F2 progeny

Trait	Year	LG	Position (cM)	Locus	LOD	LOD threshold α = 0.05% on the linkage group	LOD threshold α = 0.05% on the whole genome	Confidence interval ± 2-LOD (cM)	*R*^*2*^	Global *R*^*2*^	KW
CPW	2009	7	55.5	VVIV04	53.07	3.2	4.7	53.3–57	0.498	0.518	****
CPW	2009	18	9.2	VMC8B5	4.18	3.5		2.9–15.9	0.029		****
CPW	2010	7	55.5	VVIV04	50.13	3.4	4.5	53–57	0.446	0.537	****
CPW	2010	18	9.2	VMC8B5	12.09	3.5		4.5–13.6	0.086		****
IL	2009	7	55.5	VVIV04	36.81	3.2	4.8	52.8–56.8	0.271	0.565	****
IL	2009	14	62.1	VVIN94	20.27	3.2		59.1–64.3	0.129		****
IL	2009	18	9.2	VMC8B5	19.41	3.5		5.8–12	0.137		****
IL	2010	7	55.5	VVIV04	46.33	3.0	4.6	52.7–59.6	0.330	0.625	****
IL	2010	14	62.1	VVIN94	25.12	3.3		59.3–63.8	0.148		****
IL	2010	18	9.2	VMC8B5	19.92	3.3		5.8–12.2	0.122		****

For each trait is described, the linkage group (LG) where the QTL was identified, position on the map, name of the closest locus to the logarithm of the odds (LOD) peak, LOD value, LOD threshold on the linkage group and on the whole genome with α = 0.05%, confidence interval, phenotypic variance explained by the QTL (*R*^*2*^), global variance explained by all the QTLs detected for one trait (Global *R*^*2*^), and significant degree according to non-parametric Kruskal-Wallis test (KM)

*CPW* Cane pruning weight; *IL* internode length. Statistical significance: ****, *p* < 0.0001

For CPW and IL traits, the major QTL was detected on LG7 and explained in 2009 between 27.1 and 49.8% of the phenotypic variance. A second QTL was also identified for both CPW and IL on LG18 and explained in 2009 from 2.9 to 13.7% of the phenotypic variance. An additional QTL explaining 12.9% in 2009 and 14.8% in 2010 of the phenotypic variance was detected for IL trait and located on LG14.

**Fig. 4:**
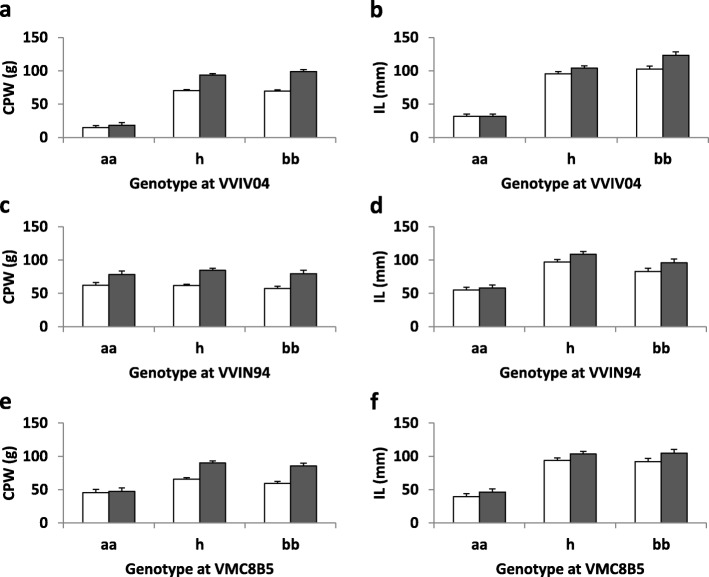
Relationships between the genotypes at the VVIV04, VVIN94 and VMC8B5 loci and the cane pruning weight (CPW) and internode length (IL) traits. The CPW (**a**, **c**, **e**) and the IL (**b**, **d**, **f**) were expressed in g and in mm respectively (white bars 2009, grey bars 2010). Vertical bars indicate standard errors. aa, homozygous with CS allele; h, heterozygous; bb, homozygous with RGM allele

At the VVIV04 closest locus of major QTLs peaks on the LG7, an ‘aa’ genotype indicated a negative impact of the CS allele ‘a’ on shoot development when this allele is homozygous (*P* < 0.0001) (Fig. 4). The ‘aa’ genotype was also deleterious for CPW and IL at the other loci (*P* < 0.001) except for CPW at the VVIN94 locus (*P* = 0.49 in 2009, *P* = 0.51 in 2010) (Fig. 3 c-f).

Based on the physical map, a total of 250, 122 and 787 genes were located within the flanking markers of the ±2-LOD confidence intervals of QTLs on LG7, LG14 and LG18 respectively.

### Genes differentially expressed between the dwarfed and normal individuals

The transcriptomes of young shoots (leaves and stems) of five normal and five dwarfed plants of the population CSxRGM_F2 were analysed using whole genome microarrays and the abundance of six transcripts was confirmed by qPCR (Additional file 5). Forty-four transcripts were up-regulated, and 8 transcripts were down-regulated in the dwarfed plants (log2 fold change > 1, *p* < 0.05 adjusted with the Holm method) (Table 2).

**Table 2:** Transcripts differentially expressed in the shoot tips between normal and dwarfed individuals of the *Vitis vinifera* cv. Cabernet-Sauvignon x *V. riparia* cv. Gloire de Montpellier F2 population (CS x RGM_F2)

	Expression level (log2)						
Microarray identifier	Mean Dwarfed	Mean Normal	Dwarf - Normal	*p* value adjusted with Holm	Gene identifier V2	Gene name	CRIBI annotation
CHRUN_JGVV1308_2_T01	5.2	11.1	−6.0	0.00	VIT_200s1308g00020		upf0308 protein chloroplastic-like
CHRUN_JGVV566_1_T01	5.0	10.4	−5.4	0.00	VIT_200s0566g00010		upf0308 protein chloroplastic-like
CHR7_JGVV31_29_T01	5.3	10.6	−5.4	0.00	VIT_207s0031g00320	CLF/CURLY LEAF	polycomb protein ez1 set domain protein
CHR7_JGVV31_32_T01	6.2	11.3	−5.1	0.00	VIT_207s0031g00350	CCoAOMT	caffeoyl-coa o-methyltransferase
CHR7_JGVV31_30_T01	6.2	10.7	−4.5	0.00	VIT_207s0031g00330		pi-plc x domain-containing protein at5g67130-like
CHR7_JGVV31_31_T01	5.7	9.5	−3.8	0.00	VIT_207s0031g00340		f-box protein
CHR7_JGVV5_241_T01	5.9	8.9	−3.0	0.03	VIT_207s0005g02490	CYP709B2	cytochrome p450
CHR5_JGVV77_76_T01	5.6	7.5	−1.9	0.03	VIT_205s0077g01020		probable n-acetyltransferase hookless 1
CHR7_JGVV129_32_T01	11.3	10.3	1.0	0.05	VIT_207s0129g00290		Formamidase
CHR17_JGVV0_115_T01	9.0	7.8	1.1	0.01	VIT_217s0000g09080	MYB50 MYB55 MYB86	hypothetical protein r2r3-myb transcription
CHR2_JGVV87_15_T01	12.3	11.1	1.2	0.05	VIT_202s0087g00840	ABCG14	white-brown-complex abc transporter family
CHR8_JGVV7_740_T01	11.1	9.9	1.2	0.03	VIT_208s0007g01180		receptor protein kinase 1-like
CHR8_JGVV40_125_T01	6.2	5.0	1.2	0.02	VIT_208s0040g02020	FLA11	fasciclin-like arabinogalactan protein 11-like
CHR13_JGVV19_208_T01	11.6	10.0	1.5	0.02	VIT_213s0019g03130 VIT_213s0019g03120	UGT85A2/ UGT85A1	udp-glycosyltransferase 85a1
CHR13_JGVV106_5_T01	9.9	8.3	1.5	0.03	VIT_213s0106g00060		ankyrin repeat-containing
CHR6_JGVV9_33_T01	12.7	11.1	1.6	0.04	VIT_206s0009g03450	LPR1	lateral root primordium protein
CHR16_JGVV22_167_T01	6.6	5.0	1.6	0.01	VIT_216s0022g00560		paired amphipathic helix protein sin3
CHR7_GSVIVT00000186001_T01	9.1	7.5	1.6	0.03	VIT_207s0129g00750		isoflavone 2 –hydroxylase
CHR19_JGVV85_28_T01	9.0	7.3	1.8	0.04	VIT_219s0085g00950	NAC028	nac domain ipr003441
CHR3_JGVV17_56_T01	8.8	7.0	1.8	0.00	VIT_203s0017g01010	AMC1/Metacaspase-1	hypothetical protein
CHR18_RANDOM_JGVV82_56_T01	8.2	6.3	1.9	0.01	VIT_218s0001g04830	TPS21/terpene synthase 21	beta-caryophyllene synthase
CHR4_JGVV43_22_T01	7.1	5.0	2.1	0.02	VIT_204s0043g00300		hypothetical protein tpx2 (targeting protein for xklp2) family protein
CHR1_JGVV150_30_T01	8.1	6.1	2.1	0.05	VIT_201s0150g00300	GH3	indole-3-acetic acid-amido synthetase
CHR4_GSVIVT00035889001_T01	7.9	5.7	2.2	0.05	VIT_204s0008g05790		hypothetical protein
CHR7_JGVV129_29_T01	10.6	8.4	2.2	0.02	VIT_207s0129g00290		formamidase
CHRUN_GSVIVT00006381001_T01	8.7	6.5	2.3	0.04	VIT_200s0358g00020		hypothetical protein
CHR13_RANDOM_JGVV112_22_T01	11.2	8.9	2.3	0.01	VIT_213s0067g00620		bifunctional dihydroflavonol 4-reductase flavanone 4-reductase
CHR8_JGVV40_238_T01	11.8	9.4	2.5	0.04	VIT_208s0040g00820	CYP94D2	cytochrome p450
CHR6_JGVV4_547_T01	11.1	8.5	2.6	0.00	VIT_206s0004g02580	BLH8	bel1 homeotic hypothetical protein
CHR2_JGVV25_23_T01	10.6	7.9	2.7	0.00	VIT_202s0025g00250	SP1L5/SPIRAL1-like 5	nitrilase-associated protein
CHR16_PDVV115_38_T01	8.7	5.9	2.8	0.00	VIT_216s0115g00410		hypothetical protein
CHR14_GSVIVT00030939001_T01	8.6	5.8	2.8	0.00	VIT_214s0006g02160		probable s-adenosylmethionine-dependent methyltransferase at5g37990
CHR8_JGVV40_281_T01	10.4	7.5	2.9	0.02	VIT_208s0040g00380		probable s-acyltransferase at5g05070-like
CHR15_JGVV24_108_T01	9.4	6.5	2.9	0.00	VIT_215s0024g00440		disease resistance protein rga3-like
CHR14_JGVV83_93_T01	8.5	5.5	3.0	0.00	VIT_214s0083g01050	SEP1	mads-box protein
CHR8_JGVV7_766_T01	11.9	8.9	3.0	0.01	VIT_208s0007g00890		tropinone reductase homolog at1g07440
CHR7_JGVV31_36_T01	8.4	5.3	3.0	0.02	VIT_207s0031g00400		zinc finger protein
CHR12_JGVV35_122_T01	8.3	5.1	3.3	0.01	VIT_212s0035g00900	JAZ12/jasmonate-zim-domain protein 12	protein tify 3b
CHR5_GSVIVT00029081001_T01	8.6	4.9	3.7	0.00	VIT_205s0051g00880		hypothetical protein
CHRUN_GSVIVT00003451001_T01	8.2	4.4	3.8	0.00	VIT_200s0193g00010		hypothetical protein
CHR19_JGVV15_10_T01	9.2	5.3	3.8	0.00	VIT_219s0015g00100	CYP71B7	cytochrome p450
CHR17_JGVV0_480_T01	9.1	5.2	3.9	0.00	VIT_217s0000g05000	SEP2	sepallata1-like protein
CHR14_JGVV108_72_T01	10.2	6.1	4.1	0.00	VIT_214s0108g00760	MIF2A	zf-hd homeobox protein at4g24660-like
CHR1_JGVV11_153_T01	9.4	5.2	4.2	0.01	VIT_201s0011g05100	MLP34	major latex
CHR10_JGVV3_187_T01	13.7	9.4	4.3	0.00	VIT_210s0003g02070	AG SHP1	agamous-like protein
CHRUN_JGVV193_1_T01	9.9	5.6	4.3	0.00	VIT_200s0193g00020		hypothetical protein
CHR17_JGVV0_372_T01	10.3	5.7	4.5	0.00	VIT_217s0000g06200	MIF1	zf-hd homeobox protein at4g24660-like
CHR15_JGVV24_104_T01	10.0	5.0	4.9	0.00	VIT_215s0024g00480	PP2-A1	protein phloem protein 2-like a1
CHR12_JGVV142_18_T01	12.5	7.0	5.5	0.00	VIT_212s0142g00360	SHP1	agamous-like protein
CHR14_JGVV81_14_T01	11.9	6.2	5.7	0.00	VIT_214s0081g00670	AHL19, AT-hook motif nuclear-localized protein 19	dna-binding protein escarola-like
CHR14_JGVV108_75_T01	12.0	6.0	6.0	0.00	VIT_214s0108g00810	MIF2B	zf-hd homeobox protein at4g24660-like
CHR1_JGVV10_295_T01	13.1	5.7	7.4	0.00	VIT_201s0010g03900	SEP3	transcription factor

Log2 fold change > 1, *p* value < 0.05 adjusted with Holm, *n* = 5

Some of the most strongly up-regulated transcripts were MADS box transcription factors: *VIT_210s0003g02070*, the grapevine orthologue of *A. thaliana AGAMOUS SHATTERPROOF1* (*VviAG SHP1*), *VIT_214s0083g01050* the grapevine orthologue of *A. thaliana SEPALLATA1* (*VviSEP1*), *VIT_201s0010g03900* the grapevine orthologue of *AtSEP3*, *VIT_212s0142g00360* the grapevine orthologue of *A. thaliana SHATTERPROOF1* (*VviSHP1*) and *VIT_217s0000g05000* (*VviSEP2*). A further 7 transcription factors were up-regulated in the dwarfed plants including one MYB transcription factor (*VIT_217s0000g09080*, *VviMYB50*/*VviMYB55*/*VviMYB86*), three zinc finger homeobox domain transcription factors, *MINI ZINC FINGER1* (*VIT_217s0000g06200, VviMIF1*), *VviMIF2B* (*VIT_214s0108g00810*) and *VviMIF2A* (*VIT_214s0108g00760*), and one BEL1-related homeobox transcription factor (*VIT_206s0004g02580*, *VviBLH8*). *VviMIF2A* and *VviMIF2B* genes are located on chromosome 14 within the ±2-LOD confidence interval of the QTL for IL. The only transcript differentially expressed between the dwarf and normal plants within the ±2-LOD confidence interval of the QTL for CPW and IL on chromosome 18 was a beta-caryophyllene synthase (*VIT_218s0001g04830,* TPS21/terpene synthase 21).

Four of the most strongly down-regulated transcripts are from genes which are contiguously located on chromosome 7, within the confidence interval of the QTLs for IL and CPW. Low hybridization signals, not different of background noise, were detected for these four genes demonstrating an absence of expression. These four genes are a SET domain-containing protein that is the orthologue of *A. thaliana* CURLY LEAF (*VIT_207s0031g00320*, *VviCLF*), a gene belonging to the phospholipase C-like phosphodiesterases superfamily (*VIT_207s0031g00330*), an F-box family protein (*VIT_207s0031g00340*) and a caffeoyl-CoA *O*-methyltransferase (*VIT_207s0031g00350*, *VviCCoAOMT*). This result combined with the ‘aa’ allelic form of VMC1A12 and VVIV04 suggested the presence of a deletion in CS genome.

### An 84,482 bp deletion was identified on chromosome 7 of dwarfed genotypes

The BAC end sequences of eight CS BAC clones were used to select in silico clones surrounding the confidence interval of the QTLs for IL and CPW on LG7. After digestion, an estimation of the insert size of each BAC was done. Two BAC clones, *VVCS1H006A20* and *VVCS1H018A11*, without and with deletion, were selected for a complete PacBio sequencing. According to in silico analyses of BAC end sequence positions, the insert size of BAC clone *VVCS1H006A20* was evaluated as 140 kb, the expected size. However, the insert size of the *VVCS1H018A11* clone should have a length of 236.1 kb, but the digestion of this clone revealed an insert size of only 145 kb, suggesting a deletion of approximately 91.1 kb.

After PacBio sequencing, the length of the insert sequences of the BAC clones *VVCS1H006A20* and *VVCS1H018A11* was 138,300 bp and 145,215 bp respectively. These sequences were compared to 12X.v2 genome sequence (Additional files 6 and 7). The insert sequence of *VVCS1H006A20* aligned from 22,790,285 to 22,944,987 bp of the chromosome 7 without large deletions and genome rearrangements. The insert sequence of *VVCS1H018A11* aligned from 22,749,398 to 24,724,688 bp of the chromosome 7 sequence. Two large deletions of 13,084 bp and 84,482 bp were shown (Additional file 8). The first deletion was located between 22,843,538 and 22,856,622 bp on the chromosome 7. Within this chromosomal region, only one potentially expressed gene without known function, *VIT_207s0031g00285*, was located. No probe of this gene was present on the microarray. The second deletion was observed between 22,863,166 and 22,947,648 bp. Within this chromosomal region, 10 potentially expressed genes were located and among them the four not expressed genes previously described (Additional file 8). The expression of the other 6 potentially expressed genes was not detected in either the normal or dwarfed plants in the tissue studied as for *VIT_207s0031g00285* (data not shown). This deletion was replaced by an insertion of a 5587 bp-long sequence which was at 26% composed with (TTA)n simple repeats of satellite DNA. We discovered two transpositions of about 600 bp within the region of the first deletion. The related sequences were composed of AT-rich satellite DNA and located between 22,868,133 and 22,868,768 bp and between 24,724,075 and 24,724,688 bp on the chromosome 7 of the 12X.v2 grape genome. The first transposition was also associated with an inversion (Additional files 7 and 8). Based on RepeatMasker2.1 results, partial direct repeats were present near the deletion junction regions.

### Presence of the 84,482 bp deletion in grapevine cultivars

The presence of the deletion of 84,482 bp on chromosome 7 was confirmed in the dwarfed individuals by PCR analysis using primers that flank the predicted deletion site in CS BAC clone *VVCS1H018A11*. As expected, DNAs from BAC clone *VVCS1H018A11*, dwarfed genotypes or CS cultivar produced a 782 bp PCR product, while DNA from normal individuals with a homozygous ‘bb’ genotype at the VVIV04 locus failed to produce any PCR products because the DNA fragment between both primers was too long to be amplified.

The presence of the deletion named ‘Delchr7’ within the genome of 51 *Vitis vinifera* cultivars was investigated thanks to the PCR-based marker (Additional file 9). The deletion was found in heterozygous form in the genome of only three cultivars: Sauvignon, CS and Arinarnoa.

## Discussion

### Three loci explain 62.5% of the total variance in IL

Using an interspecific *V. vinifera x V. riparia* F2 population with 17% of dwarfed and abnormal individuals, we identified three loci related to IL on LG7, 14 and 18, explaining 33.0, 14.8 and 12.2% of the phenotypic variance, respectively. The transcriptomic analysis suggested that four genes within the interval of the QTL on LG7 were absent in the genome of dwarfed plants, which has been confirmed by BAC clone sequencing.

To the best of our knowledge, the only well characterized grapevine dwarf is the gibberellin insensitive *Vvigai1* mutation identified in *V. vinifera* cv. Pinot meunier [3], which does not co-locate with any of our QTLs. In other species, studies of the genetic architecture of dwarfism and IL suggest that these traits are mainly under the control of genes belonging to two functional categories. Firstly and most frequently observed, genes coding for proteins are involved in hormone metabolism. For example, the *cp* locus that confers a dwarf phenotype to cucumber and was shown by fine mapping to co-locate with a cytokinin oxydase gene [21]; the “Rinrei” mutant of faba bean, impaired on brassinosteroid biosynthetic gene *bdd1,* which codes for a C-24 sterol reductase [22]; and the *GmDW1* locus of soybean, which corresponds to an ent-kaurene synthase, one of the early steps of the gibberellin biosynthetic pathway [23]. Secondly, genes coding transcription factors involved in the regulation of shoot development and architecture, such as *Reduced height* in wheat, that codes for a DELLA transcription factor [24]; or the *dil1* locus in maize that was shown by map-based cloning to correspond to AP2-like gene [25]. None of the above-mentioned genes were found in the confidence intervals of our QTLs.

### Four genes located inside the 84,482 bp deletion of chromosome 7 were not expressed in the dwarfed plants

Among 11 genes deleted on chromosome 7 of the dwarfed plants, nine are present on the microarray and two were quantified by qPCR. Of these 11 genes, the transcripts of four genes were not expressed in the plants with a dwarfed phenotype, but were expressed in normal individuals; they were *VviCLF*, *VIT_207s0031g00330*, *VIT_207s0031g00340* and *VviCCoAOMT*. CLF is a well-described developmental regulator that participates in transcriptional repression via methylation of histone H3 lysine 27 (H3K27) in the polycomb repressive complex 2 in *A. thaliana* [26]. Mutants of *CLF* show early flowering and curled leaves, and this phenotype is largely caused by the mis-expression of the floral homeotic gene *AG* in leaves [27]. In wild type plants, *AG* is expressed only in flowers where it specifies the identity of stamens and carpels. The absence of *VviCLF* in the dwarfed grapevine plants studied here was associated with plants with curled, small leaves and the up-regulation of *VviAG SHP1* expression in vegetative shoot tissue. In addition to the mis-expression of *VviAG SHP1*, a number of other floral homeotic genes were up-regulated in the dwarfed plants such as *VviSEP1*, *VviSEP2*, *VviSEP3* and *VviSHP1*. The mis-expression of various flower identity and flowering time control genes have also been reported in *A. thaliana clf* and *ag* mutants, such as the mis-expression of *AtAP2*, *AtSHP1*, *AtSHP2*, *AtSEP3*, *FLOWERING LOCUS T* and *FLOWERING LOCUS C* [26–28]. This could suggest that VviCLF protein has similar functions in grapevine to that of AtCLF in terms of floral gene repression in vegetative tissues in *A. thaliana*.

Reproductive development in grapevine differs significantly from that of annual plants such as *A. thaliana.* In temperate regions, floral initiation occurs in the spring/summer in latent buds and these buds remain dormant over the following winter. Mature flowers develop from immature primordia at bud break. *VviCLF* is highly expressed in latent bud during the flower initiation and at bud break, and is not expressed during the dormant period [29]. Like its *A. thaliana* orthologue, *VviCLF* is also highly expressed in vegetative tissue such as leaves and tendrils [29]. Potentially the mis-expression of floral homeotic genes in the dwarfed plants could be responsible for the absence of flowers in these plants. *VIT_207s0031g00330* was absent from the dwarfed plants, VIT_207s0031g00330 is a phospholipase C-like phosphodiesterases superfamily protein. Phospholipase C-like phosphodiesterases are intracellular enzymes with important roles in signal transduction processes [30], but the function of most proteins remains unknown. VIT_207s0031g00340 is an F-box protein; these proteins generally provide substrate specificity for Skp1-Cullin-F-Box complexes that direct protein degradation via the ubiquitin-26S proteasome pathway [31]. F-box proteins are responsible for the regulation of a wide range of biological processes and there are 156 F-box proteins present in the grapevine genome [32], as such, it is difficult to assign a putative function to VIT_207s0031g00340. Caffeoyl-CoA *O*-methyltransferases have essential roles in lignin biosynthesis in both herbaceous and woody plants [33]. *A. thaliana* mutants in *CCoAOMT1* shows slightly reduced development under short-day conditions, but no visual phenotype under long days [34]. Poplar trees with reduced CCoAOMT activity exhibit no obvious visible phenotype, yet reduced lignin contents [35]. The loss of *VviCCoAOMT* in the dwarfed plants was associated with the up-regulation of expression of an arabinogalactan protein (*VIT_208s0040g02020*) and a cinnamyl alcohol dehydrogenase (*VIT_213s0067g00620*), a similar result has been observed in *ccoaomt1* mutants of *A. thaliana* (an arabinogalactan protein and a cinnamoyl-CoA reductase were up-regulated) [36]. This may suggest that the loss of a CCoAOMT triggers some degree of cell wall modification in the dwarfed plants which is similar to that of *A. thaliana ccoaomt* mutants.

### VviMIF2A and VviMIF2B are up-regulated in the dwarfed plants and are located within the QTL of IL on LG 14

In addition to the differential accumulation of transcripts of the floral regulators cited above, a number of transcription factors were differentially expressed between the dwarfed and normal individuals, including the up-regulation of three zinc finger homeobox transcription factors *VviMIF1*, *VviMIF2A* and *VviMIF2B*. MIF1 is known to regulate plant hormone signalling pathways and MIF1 over-expressing *A. thaliana* plants (*35S::MIF1*) show dwarf phenotypes with reduced apical dominance, dark-green leaves, curled leaves, altered flower morphology, poor fertility and spoon like cotyledons [37]. *35S::MIF2* and *35S::MIF3* lines have similar visible phenotypes to those of 35S::MIF1 [38]. It was also suggested that MIF1 and MIF3 have roles in meristem formation as severe *35S::MIF1* or *35S::MIF3* plants have ectopic shoot meristems on leaf margins and develop ovules along the edges of sepals [38]. The phenotype of MIF over-expressers is similar to that of the dwarfed grapevines in this study suggesting that the increase in MIF transcription factors could also explain some of the phenotypic characteristics of the dwarfed plants. Furthermore, *VviMIF2A* and *VviMIF2B* are within the confidence interval of the QTL for IL on LG14 suggesting that they may directly control some part of the dwarfed phenotype.

### Genetic origin of the 84,482 bp deletion on chromosome 7 of dwarfed plants

**Fig. 5:**
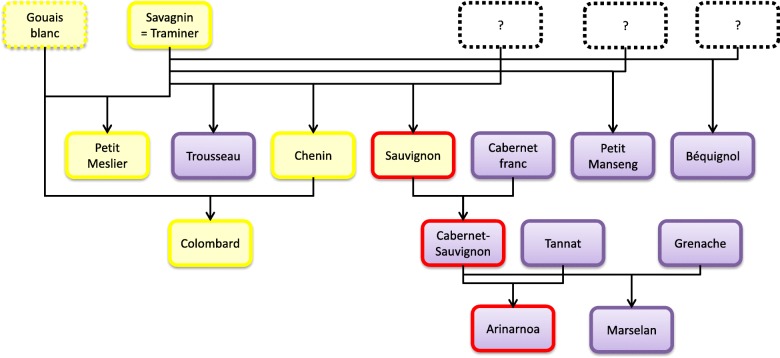
Genetic origin of the deletion ‘Delchr7’. The presence of the deletion ‘Delchr7’ was evaluated within the parentage and kin group of Savagnin cultivar. White and red cultivars are shown by rectangles filled with yellow or purple respectively. The presence of the deletion ‘Delchr7’ in the genome is shown by a red outline. Dotted lines indicate that the cultivar was not studied

The 84,482 bp deletion on chromosome 7 was unique to the CS genome and other insertions/deletions were not found in the RGM genome at Delchr7 locus which was homozygous [39]. Considering that the recessive locus was given by the female *V. vinifera* parent of the F2 progeny, the presence of the 84,482 bp deletion was investigated in the genome of 51 *V. vinifera* varieties. The deletion ‘Delchr7’ was found in the genome of three of the 11 members of the kin group of Savagnin: Sauvignon, CS (which is a progeny of Sauvignon and the mother of the F2 progeny studied here), and Arinarnoa (which is a progeny of CS). Taking into account the parentage of the investigated cultivars [40–42], this shows that ‘Delchr7’ of CS came from the unknown parent of Sauvignon or was the result of meiotic events during the genetic cross between Savagnin and this unknown parent (Fig. 5). Savagnin has probably crossed with a single, unknown and probably extinct variety to give birth to the siblings Sauvignon, Trousseau and Chenin [41]. However, only Sauvignon received ‘Delchr7’ in its genome and transmitted it to its offspring. The ‘Delchr7’ locus does not appear to have a negative impact on shoot development when this locus is heterozygous (i.e. in Sauvignon, CS and Arinarnoa), thus it may be concluded that ‘Delchr7’ is a deleterious recessive locus.

In addition to the 84,482 bp deletion, another large deletion of 13,084 bp, a transposition and a transversion were identified on chromosome 7 of dwarfed plants in comparison to the 12X.v2 genomic sequence. It is known that active mobile elements can cause chromosomal rearrangements, including genomic deletion [43]. Although partial direct repeats were identified near the deletion junction regions by in silico analysis, it is not clear whether they were involved in the formation of deletions.

Repetitive DNA sequences with a variable AT-rich repeat unit were identified within the inserted sequence replacing the 84,482 bp deletion region and within the two transposition sequences. Satellite DNAs are accumulated in the heterochromatin, mainly in centromeric and subtelomeric regions. Repetitive DNA sequences have developmental, cellular, and cytoplasmic effects and play a role in chromosomal recombination [44]. They are involved in several changes, such as transposition, segmental duplications and mechanisms based on rolling-circle replication of extrachromosomal circular DNAs and reinsertion [45]. Thus, the chromosomal rearrangements evidenced on chromosome 7 of CS x RGM_F2 dwarfed plants could be a consequence of the presence of repetitive DNA sequences.

## Conclusions

The combination of genetic and transcriptomic analyses facilitated the identification of a major deletion on chromosome 7 of CS which, when homozygous, appears largely responsible for dwarfing in an interspecific *V. vinifera* x *V. riparia* F2 population. Other QTLs on LGs 14 and 18 were also identified for IL trait. The large deletion of 84,482 bp on chromosome 7 of CS encompasses 10 genes, among them *VviCLF*, a major developmental regulator that putatively suppresses the expression of floral homeotic genes in vegetative tissues. The deletion was found in relatives of CS such as Sauvignon, but is not widely distributed in grapevine cultivars. This work is the first molecular characterization of a deleterious recessive locus in grapevine potentially explaining dwarfed phenotypes in backcrosses or F2 populations with CS and its relatives. The PCR-based molecular marker ‘DelChr7’ defined in this study could now be used to track for the presence of the deletion in Sauvignon and CS based progenies, in order to limit the risk of appearance of abnormalities in subsequent crosses.

## Methods

### Plant material

The F2 population used in this study, named CS x RGM_F2, consisted of 337 individuals resulting from the inter-specific cross of *V. vinifera* cv. CS x *V. riparia* cv. RGM. This F2 population, developed in 2004 at INRA Bordeaux, France, derived from the self-fertilization of the F1_148 individual of the F1 CS X RGM1995–1 population [9]; itself obtained in 1995 at INRA Bordeaux, France, by a cross between *V. vinifera* cv. CS and *V. riparia* cv. RGM [9].

The F1_148 individual and the CS x RGM_F2 population were maintained in pots in a naturally illuminated and semi-regulated greenhouse, with one plant per genotype. *V. vinifera* cultivars and *V. riparia* cv. RGM were present in vineyards at INRA Bordeaux, France. For all genotypes used in this study, leaves were collected in greenhouse and in vineyards according to institutional guidelines and directly used for total nucleic acid extraction. The identification of the *V. vinifera* cultivars and *V. riparia* cv. RGM was done by the Institut Français de la Vigne et du Vin, France, by SSR markers. No permissions were required to obtain this plant material. SSR markers were also used at INRA Bordeaux, France, for the identification of the F1_148 individual [9] and the CS x RGM_F2 population.

### Total nucleic acid extraction

Leaf samples (approximately 0.3 g fresh weight) were ground with a rolling grinder (HOMEX, Bioreba) in 5 mL of metabisulfite buffer containing 0.2 M Tris-HCl pH 8.0, 70 mM EDTA pH 8.0, 2 M NaCl and 20 mM sodium metabisulfite. Aliquots of leaf extracts (0.5 mL) were placed in tubes and 450 μL of HATMAB buffer (2% HATMAB w/v, 1.4 M NaCl, 20 mM EDTA pH 8.0, 100 mM Tris-HCl pH 8.0) was added. Tubes were vortexed vigorously, incubated for 1 h at 65 °C and centrifuged at 1600 *g* for 25 min at 4 °C. Five hundred μL of the supernatants were recovered and transferred to new tubes, followed by the addition of 450 μL chloroform-octanol (24:1). The mixture obtained was vortexed and then incubated on ice with intermittent shaking. The tubes were centrifuged at 1600 *g* for 20 min at 4 °C. Three hundred μL of the supernatant were recovered, and added with 150 μL 10 M ammonium acetate and 300 μL isopropanol. Tubes were transferred at − 20 °C for 25 min. Total nucleic acids were pelleted by centrifugation at 1600 *g* for 25 min at 4 °C. Supernatants were removed and nucleic acid pellets were washed with ice cold 70% EtOH, air dried, and dissolved in 200 μL 0.1X TE buffer.

### Choice of molecular markers

SSR markers were mainly chosen from the markers used to construct the map of the F1 CS X RGM1995–1 population [9]. In addition, 46 new SSR markers were designed using the grape genome 12X sequence (http://www.genoscope.cns.fr/externe/GenomeBrowser/Vitis/, Additional file 1). Primers were designed with PRIMER0.5 software (Whitehead Institute for Biomedical Research). A new insertion-deletion marker *VvOMT2–2* was also designed based on the *VviOMT2* gene sequence [46]. A pair of primers, VvOMT2_2F:5′-AACTTTGCAGATGATAATCGAGG-3′ and VvOMT2_2R:5′-ATGGATTCGACATTGAGAAAATG-3′, were used to detect the presence an insertion-deletion of 7 bp in the 3’UTR of *VviOMT2* gene (*VIT_12s0059g01750*).

### Amplification of SSR molecular makers

All PCR reactions were performed in 15 μL reaction volume containing: 10 ng of template DNA, 1x PCR reaction buffer, 2 mM MgCl_2_, 0.2 mM of each dNTP, 0.2 μM dye conjugated M13 primer, 0.05 μM M13 tailed SSR forward primer, 0.2 μM SSR reverse primer and 0.025 U JumpStart™ *Taq* DNA Polymerase (Sigma). All PCR forward primer oligonucleotides were tailed on their 5’end with one of the following M13 forward sequences: A13, CACGACGTTGTAGGACCAC, B13, CACGTTCTGGAACATCGAC or C13, CACGCACTTGACGAAGGAC. Fluorescent dye (PET, NED, VIC or 6-FAM) was incorporated in amplicons by including a 5′ dye-labelled M13 forward primer in the PCR. PET, NED and VIC fluorescent dyes were associated to A13, B13 and C13 respectively and FAM fluorescent dye with the three M13 forward sequences.

The PCR thermocycler conditions were the same for all primers pairs and adapted from the literature [9]: 5 min initial denaturation step at 94 °C, followed by 3 cycles of 30 s denaturation at 94 °C, 1 min 30 s annealing at 55 °C or 60 °C and 1 min extension at 72 °C, followed by 35 cycles of 30 s denaturation at 94 °C, 30 s annealing at 55 °C or 60 °C and 1 min extension at 72 °C then followed by 7 min final extension at 72 °C.

SSR markers were first tested for amplicon marker quality with DNA samples obtained from CS, RGM, F1_148 and five individuals of the F2 population. Marker allele size ranges and single loci in the expected amplicon size ranges were evaluated. Polymorphic markers were then run on the entire CS X RGM_F2 mapping population.

A Hamilton STARlet robot (HAMILTON Robotics) was used to deposit 4 μL genomic DNA of the each genotype of the CS x RGM_F2 population in 384-well PCR plates. PCR amplifications were made by multiplexing in single PCR reaction 2 to 4 markers sharing the same dye conjugated M13 primer. In a PCR reaction, markers sharing the same dye conjugated M13 primer could be only discriminated by their PCR product length.

### Marker evaluation and genotyping

PCR amplicons and GeneScan™ 600 LIZ® dye internal size standards (Life Technologies Corporation) were separated by capillary electrophoresis using ABI 3730 (Life Technologies Corporation). Markers were multiplexed by 12 or 13 per capillary channel by combining with Hamilton STARlet robot aliquots of 4–6 PCR reactions. Allele sizing was performed with ABI PRISM GENEMAPPER 4.0 software (Life Technologies Corporation) according to the manual instructions.

### Linkage analysis and mapping

The map was constructed using the software CarthaGene [47] at a logarithm of the odds (LOD) value of 5.0 and at a maximal distance threshold of 35 cM. Validation of the map obtained was done using the software JoinMap® 3.0 [48] using a Kosambi’s mapping function. The marker order obtained was checked according to the consensus map of the F1 population CS x RGM1995–1 and to the 12X genome sequence. The linkage groups (LGs) were numbered LG1–LG19, according to [17].

### Phenotypic measurements

The CPW was evaluated and the length of the third internode (IL) was measured at the end of 2009 and 2010. Shoot number per plant was two and the longest shoot was systematically chosen for measurements. Pearson correlation coefficients were evaluated using R [49].

### QTL analysis

Data normality for each quantitative trait was evaluated with Shapiro-Wilkinson test. Despite deviations from normality for each trait, data were not transformed because the interval mapping method is robust to deviations from this assumption [50]. QTL detection was performed using the raw metric measurements with MapQTL 6.0 software [51] and adapted from the literature [9]. Four statistical methods were employed: Kruskal-Wallis analysis, interval mapping, MQM and permutation test. Four was retained as the maximum number of co-factors. The minimum LOD score used for QTLs detection was three. The significant LOD threshold was calculated at 5% for the LG and for the genome-wide through 1000 permutations. The maximum LOD value was retained for QTL position and a ± 2-LOD interval for the confidence interval. Differences between the genotype at the VVIV04, VVIN94 and VMC8B5 loci, and the shoot development traits were tested for significance using R [49] by applying analysis of variance (ANOVA) followed by Tukey test (*p* value < 0.05).

### RNA extraction

Young leaves and stems were harvested and immediately snap frozen in liquid nitrogen. Total RNA was extracted using the Spectrum Plant Total RNA kit (Sigma-Aldrich) according to the manufacturer’s instructions.

### Microarray analysis

Roche Nimblegen oligonucleotide microarrays (Design 090918 Vitus exp. HX12) were used for whole genome transcriptome analysis. This microarray probe design for the 29,549 transcripts studied is based on the 12X genome assembly using the grapevine V1 gene model prediction from CRIBI (http://genomes.cribi.unipd.it/). The correspondence between probe identifiers and gene identifiers were obtained from CRIBI V2 (http://genomes.cribi.unipd.it).

The microarray hybridisations were done by the Plateforme Biopuces, Institut National des Sciences Appliquées, Toulouse, France for the 10 individuals (five with a dwarfed and five with a normal phenotype); the protocol followed was as recommended by the manufacturer.

R was used to analyze the microarray data [49] as described by [52]. The limma package was used to identify differentially expressed genes [53]; genes with absolute log_2_ fold changes > 1 and Holm corrected *p* values below 0.05 were considered significant.

### qPCR analysis

For qPCR experiments, total RNA was treated with the Turbo DNA-free kit from Ambion to remove genomic DNA contamination and the reverse transcription was done using the Superscript III kit from Invitrogen (using oligo dT primers and 1.5 μg RNA). Gene expression was analyzed with iQ Sybr Green Supermix on a Biorad CFX96 machine (primer concentration of 250 nM). The expression of genes of interest was normalised with SAND protein (*VIT_206s0004g02820)* and one additional reference gene were used to confirm the stability of expression of *VIT_206s0004g02820* (Additional file 10). Two technical replicates were used in this study. PCR efficiency for each primer pair was calculated using LinRegPCR [54].

### Plasmid DNA preparation and insert size estimation

DNA from CS bacterial artificial chromosome (BAC) clones VVCS1H006A20, VVCS1H011N07, VVCS1H012O11, VVCS1H12O17, VVCS1H018A11, VVCS1H065F12, VVC1H073F06 and VVCS1H03O10 [55] was isolated using the Nucleobond Xtra Midi Plus kit (Macherey Nagel) according to the manufacturer’s instructions with chloramphenicol selective marker (12.5 μg mL^− 1^). To estimate insert size, 150 ng of each BAC was digested with the fast NotI enzyme (Fermentas) and incubated 40 min at 37 °C. After incubation, the enzymatic digestion was transferred in a gel (0.8% agarose – TBE 0.25X) for pulse field electrophoresis performed with a Chef Mapper XA CHILLER SYSTEM 220 V (Biorad) under the following conditions: voltage of 6 V cm^− 1^, included angle of 120°, initial switch time of 5 s, final switch time of 15 s, run time of 16 h with linear ramping. Each insert size was estimated using the Genetools software (Syngene).

### Sanger sequencing of BAC extremities

Based on estimated insert sizes, Sanger sequencing reactions were completed using Big Dye Terminator chemistry v3.1 (Applied Biosystems) on plasmid DNA of CS BAC clones VVCS1H018A11 and VVCS1H006A20 (around 300 ng) following the protocol described by [56] using T7 and M13r universal primers for BAC-end sequencing. Reaction products were analysed on an ABI 3730 DNA Analyzer (Applied Biosystems) at GeT-PlaGe platform (http://get.genotoul.fr/).

### PacBio sequencing

About 1.5 μg of DNA from both BACs VVCS1H018A11 and VVCS1H006A20 were pooled and sequenced using the standard Pacific Biosciences library preparation protocol for 10 kb libraries. Each replicate was sequenced in one SMRT Cell using the P6 polymerase in combination with the C4 chemistry, according to the manufacturer’s instructions (by IGM: http://igm.ucsd.edu/genomics/).

### PacBio assembly

Assembly of the PacBio reads was performed following the HGAP workflow (https://github.com/PacificBiosciences/Bioinformatics-Training/wiki/HGAP [57]. The SMRT® Analysis (v2.3.0) software suite was used for HGAP implementation (https://github.com/PacificBiosciences/SMRT-Analysis).

Reads were first aligned by BLASR (https://github.com/PacificBiosciences/blasr; [58] against “*Escherichia coli* strain K12 substrain DH10B complete genome”. Identified *E. coli* reads and low quality reads (read quality < 0.80 and read length < 500 bp) were removed from data. Filtered reads were then preassembled to generate long sequences. The sequences obtained were filtered against vectors sequences and the Celera assembler was used to get a draft assembly. The last step of HGAP workflow was the “polishing” that significantly reduced the remaining insertions/deletions and base substitution errors in the draft assembly. The Quiver algorithm (https://github.com/PacificBiosciences/GenomicConsensus/blob/master/doc/QuiverFAQ.rst) was used to enrich the quality scores embedded in Pacific Biosciences bas.h5 files. The “polished assemblies” were identified by matching their BAC end sequences with BLAST.

### In silico analyses of BAC insert sequences

The obtained insert sequences of BAC clones VVCS1H018A11 and VVCS1H006A20 were aligned to 12X.v2 genome sequence [59] using MUMmer4 software [60]; https://github.com/mummer4/mummer). The presence of interspersed repeats and low complexity DNA sequences was evaluated using the RepeatMasker2.1 software (https://github.com/rmhubley/RepeatMasker).

### PCR-based deletion marker

A pair of primers Delchr7F:5′-GGGTTGCAACTATGGTGATGCT-3′ and Delchr7R: 5′-CACAGGCACGGGTCACTCTC-3′ were manually designed and used to detect the presence of the 84,482 bp deletion in the genomic DNA of dwarfed genotypes of CSxRGM_F2 population and in the genome of 51 *V. vinifera* cultivars (Additional file 9). All PCRs were performed in 15 μL reaction volume containing: 10 ng of template DNA, 1x PCR reaction buffer, 2 mM MgCl_2_, 0.2 mM of each dNTP, 0.2 μM of each primer, 0.025 U of JumpStart™ *Taq* DNA Polymerase (Sigma Aldrich). The PCR thermocycler conditions were 5 min initial denaturation step at 94 °C followed by 35 cycles of 30 s denaturation at 94 °C, 1 min annealing at 62 °C and 1 min extension at 72 °C, followed by 5 min final extension at 72 °C. The PCR product obtained were analysed on 1.8% agarose gel. A PCR product length of 782 bp is observed when a deletion is present in the genome of evaluated genotypes.

## Supplementary information

**Additional file 1: Table S1.** New SSR markers developed

**Additional file 2: Figure S1.** Linkage map of *Vitis vinifera* cv. Cabernet-Sauvignon x *V. riparia* c*v. riparia* Gloire de Montpellier F2 population (CS x RGM_F2). Linkage groups are named according to international consensus map. Distances are in cM Kosambi

**Additional file 3: Table S2.** Characteristics of the CS x RGM_F2 linkage map

**Additional file 4: Figure S2.** Photographs of CS x RGM_F2 population growing in greenhouse. **a** Genotypes with normal phenotypes. **b-d** Individuals with dwarfed phenotypes showing curled leaves (**e-g**)

**Additional file 5: Figure S3.** Validation of microarray data by qPCR of genes differentially expressed between dwarfed and normal individuals from CS x RGM_F2 population. **a** *VIT_207s0031g00320*; **b** *VIT_207s0031g00330*; **c** *VIT_207s0031g00340*; **d** *VIT_207s0031g00350*; **e** *VIT_214s0108g00810* and **f** *VIT_214s0108g00760*. Means and standard errors shown, *n* = 3

**Additional file 6: Table S3.** Alignment of *VVCS1H006A20* insert BAC sequence to the chromosome 7 sequence. MUMmer4 software was used to align insert BAC sequence to the chromosome 7 sequence from the 12X.v2 genome

**Additional file 7: Table S4.** Alignment of *VVCS1H018A11* insert BAC sequence to the chromosome 7 sequence. MUMmer4 software was used to align insert BAC sequence to the chromosome 7 sequence from the 12X.v2 genome

**Additional file 8: Figure S4.** The physical map of the 84,482 bp deletion on chromosome 7 based on the BAC clone *VVCS1H018A11*. Double arrows delimited the two large deletions identified of 13,084 bp and 84,482 bp respectively. All dotted lines delimited similar sequences between the chromosome 7 sequence from the 12X.v2 PN40024 reference genome and the BAC clone *VVCS1H018A11* (Additional file 7). Red dotted lines were used for transposition-inversions and green ones for transpositions. Physical positions are given in kb for the BAC clone *VVCS1H018A11* sequence and in Mb for the chromosome 7 of PN40024 genotype. The 11 genes deleted on chromosome 7 of the dwarfed plants are symbolized in blue

**Additional file 9: Table S5.** Presence of the deletion within the genome of fifty-one grapevine cultivars. -, absence of deletion. +, presence of deletion

**Additional file 10: Table S6.** Sequence and mean PCR efficiency of primers used for qPCR analysis

## Abbreviations

BAC: Bacterial artificial chromosome
cM: Centimorgan
CPW: Winter cane pruning weight
CS: *Vitis vinifera* cv. Cabernet-Sauvignon
cv.: Cultivar
IL: Internode length
LG: Linkage group
LOD: Logarithm of the odds
MQM: Multiple QTL mapping
QTL: Quantitative Trait Loci
RGM: *Vitis riparia* c*v. riparia* Gloire de Montpellier
SSR: Simple sequence repeat
