# Supplementary material for: Dissecting the control of shoot development in grapevine: genetics and genomics identify potential regulators
Source: BMC Plant Biol. 2020 Jan 29;20:43. doi: 10.1186/s12870-020-2258-0 (PMC6988314; doi:10.1186/s12870-020-2258-0)
Supplement: Supplementary file 2 — Additional file 2: Figure S1. Linkage map of Vitis vinifera cv. Cabernet-Sauvignon x V. riparia cv. riparia Gloire de Montpellier F2 population (CS x RGM_F2). Linkage groups are named according to international consensus map. Distances are in cM Kosambi [file 12870_2020_2258_MOESM2_ESM.pptx]

## Slide 1
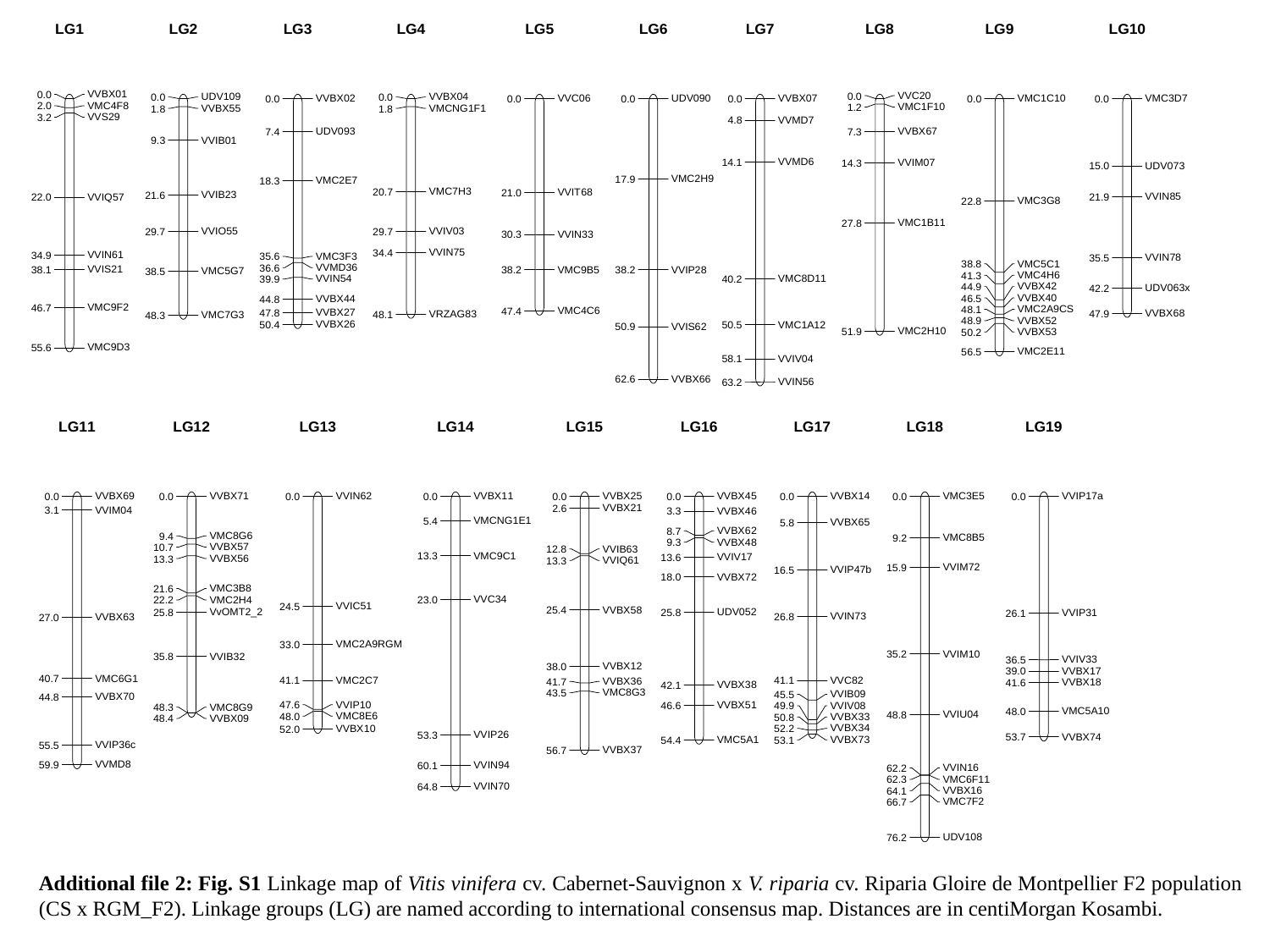

Additional file 2: Fig. S1 Linkage map of Vitis vinifera cv. Cabernet-Sauvignon x V. riparia cv. Riparia Gloire de Montpellier F2 population (CS x RGM_F2). Linkage groups (LG) are named according to international consensus map. Distances are in centiMorgan Kosambi.
