# Supplementary material for: Dissecting the control of shoot development in grapevine: genetics and genomics identify potential regulators
Source: BMC Plant Biol. 2020 Jan 29;20:43. doi: 10.1186/s12870-020-2258-0 (PMC6988314; doi:10.1186/s12870-020-2258-0)
Supplement: Supplementary file 4 — Additional file 4: Figure S2. Photographs of CS x RGM_F2 population growing in greenhouse. a Genotypes with normal phenotypes. b-d Individuals with dwarfed phenotypes showing curled leaves (e-g) [file 12870_2020_2258_MOESM4_ESM.pptx]

## Slide 1
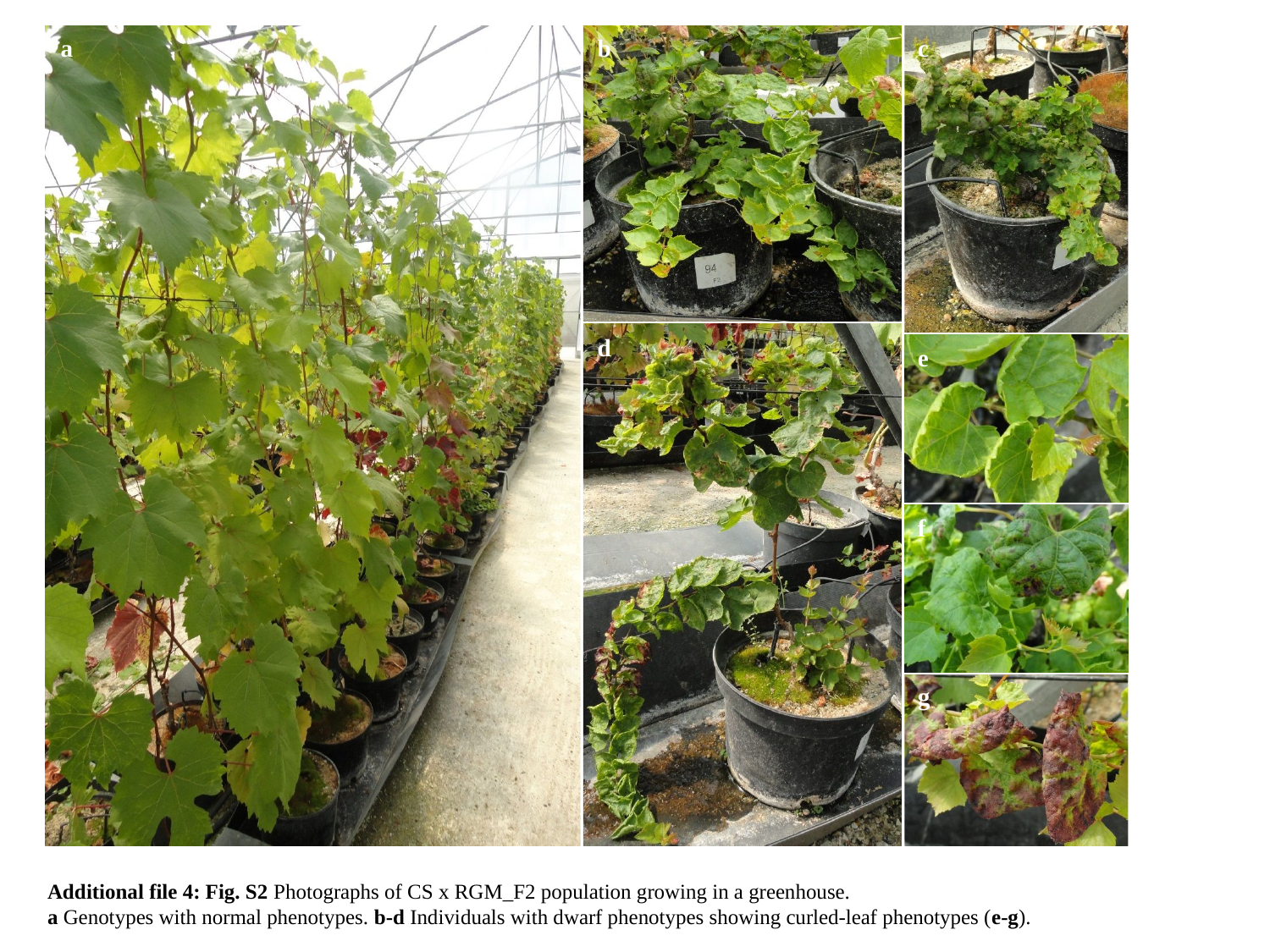

a
b
c
d
e
f
g
Additional file 4: Fig. S2 Photographs of CS x RGM_F2 population growing in a greenhouse.
a Genotypes with normal phenotypes. b-d Individuals with dwarf phenotypes showing curled-leaf phenotypes (e-g).
