# Supplementary material for: Dissecting the control of shoot development in grapevine: genetics and genomics identify potential regulators
Source: BMC Plant Biol. 2020 Jan 29;20:43. doi: 10.1186/s12870-020-2258-0 (PMC6988314; doi:10.1186/s12870-020-2258-0)
Supplement: Supplementary file 5 — Additional file 5: Figure S3. Validation of microarray data by qPCR of genes differentially expressed between dwarfed and normal individuals from CS x RGM_F2 population. a VIT_207s0031g00320; b VIT_207s0031g00330; c VIT_207s0031g00340; d VIT_207s0031g00350; e VIT_214s0108g00810 and f VIT_214s0108g00760. Means and standard errors shown, n = 3 [file 12870_2020_2258_MOESM5_ESM.pptx]

## Slide 1
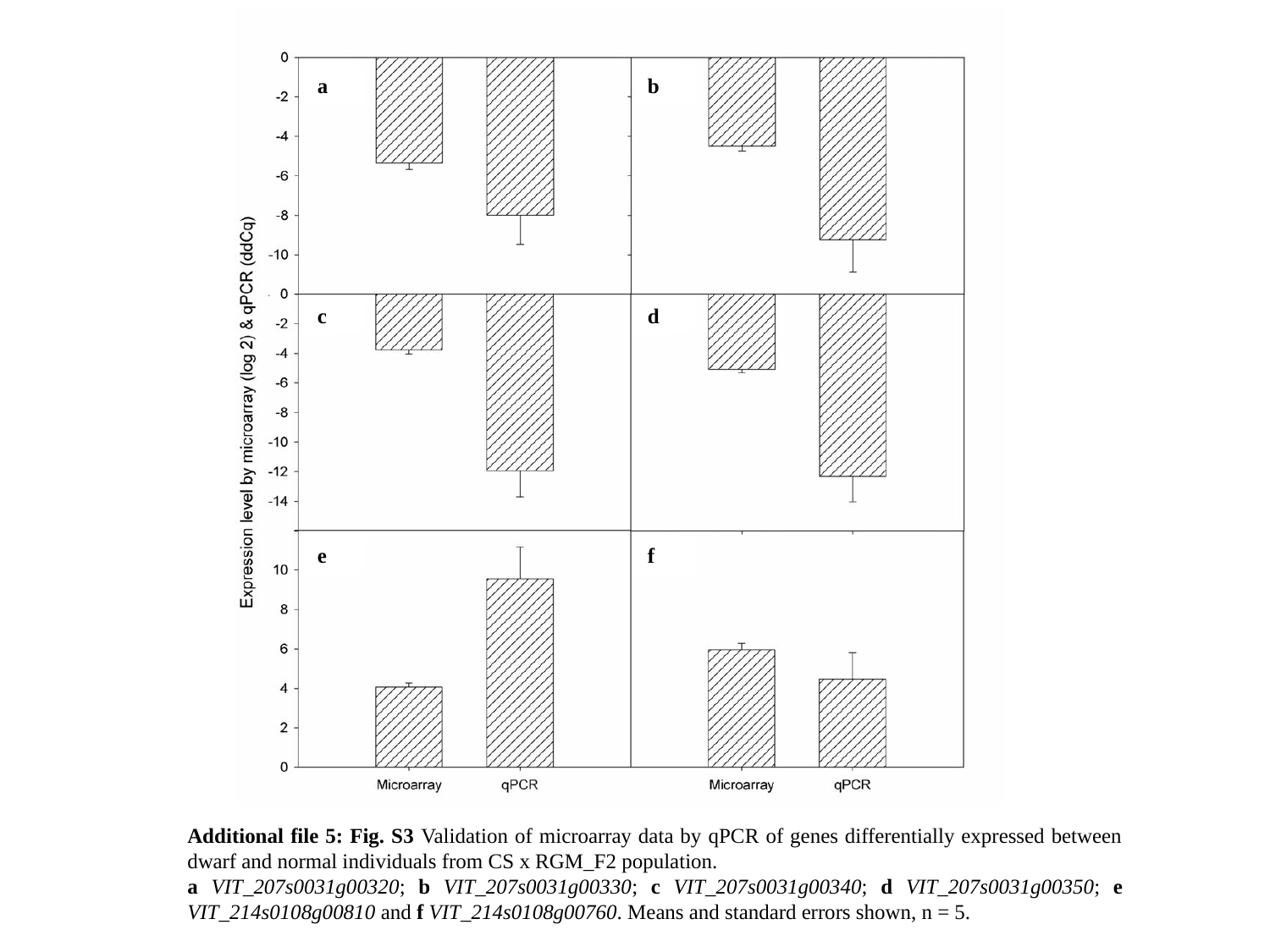

a
b
c
d
e
f
Additional file 5: Fig. S3 Validation of microarray data by qPCR of genes differentially expressed between dwarf and normal individuals from CS x RGM_F2 population.
a VIT_207s0031g00320; b VIT_207s0031g00330; c VIT_207s0031g00340; d VIT_207s0031g00350; e VIT_214s0108g00810 and f VIT_214s0108g00760. Means and standard errors shown, n = 5.
