# Supplementary material for: Dissecting the control of shoot development in grapevine: genetics and genomics identify potential regulators
Source: BMC Plant Biol. 2020 Jan 29;20:43. doi: 10.1186/s12870-020-2258-0 (PMC6988314; doi:10.1186/s12870-020-2258-0)
Supplement: Supplementary file 8 — Additional file 8: Figure S4. The physical map of the 84,482 bp deletion on chromosome 7 based on the BAC clone VVCS1H018A11. Double arrows delimited the two large deletions identified of 13,084 bp and 84,482 bp respectively. All dotted lines delimited similar sequences between the chromosome 7 sequence from the 12X.v2 PN40024 reference genome and the BAC clone VVCS1H018A11 (Additional file 7). Red dotted lines were used for transposition-inversions and green ones for transpositions. Physical positions are given in kb for the BAC clone VVCS1H018A11 sequence and in Mb for the chromosome 7 of PN40024 genotype. The 11 genes deleted on chromosome 7 of the dwarfed plants are symbolized in blue [file 12870_2020_2258_MOESM8_ESM.pptx]

## Slide 1
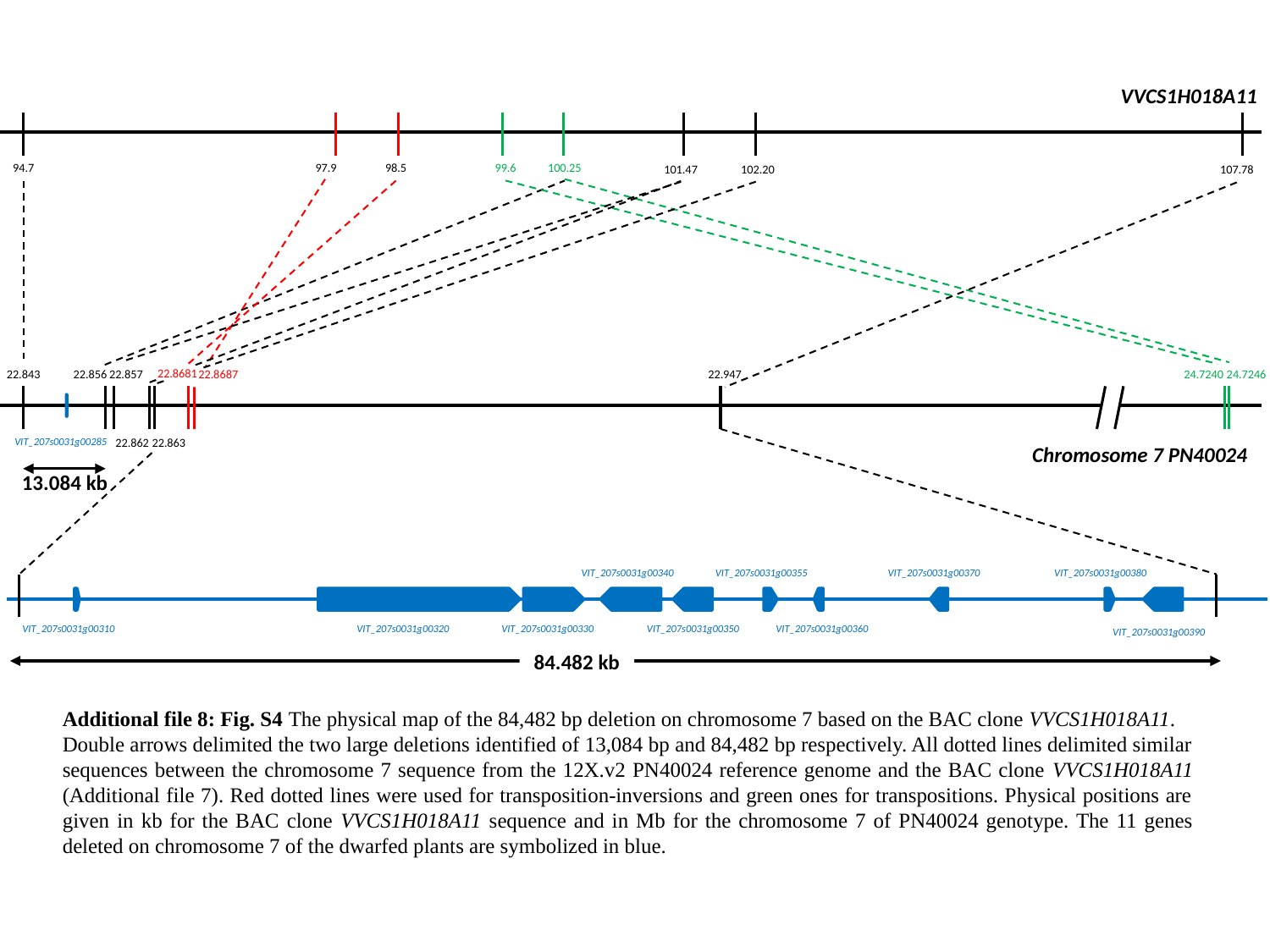

VVCS1H018A11
94.7
97.9
99.6
100.25
98.5
102.20
107.78
101.47
24.7240
24.7246
22.843
22.856
22.857
22.8687
22.947
22.8681
VIT_207s0031g00285
22.862
22.863
Chromosome 7 PN40024
13.084 kb
VIT_207s0031g00380
VIT_207s0031g00340
VIT_207s0031g00355
VIT_207s0031g00370
VIT_207s0031g00310
VIT_207s0031g00320
VIT_207s0031g00330
VIT_207s0031g00350
VIT_207s0031g00360
VIT_207s0031g00390
84.482 kb
Additional file 8: Fig. S4 The physical map of the 84,482 bp deletion on chromosome 7 based on the BAC clone VVCS1H018A11.
Double arrows delimited the two large deletions identified of 13,084 bp and 84,482 bp respectively. All dotted lines delimited similar sequences between the chromosome 7 sequence from the 12X.v2 PN40024 reference genome and the BAC clone VVCS1H018A11 (Additional file 7). Red dotted lines were used for transposition-inversions and green ones for transpositions. Physical positions are given in kb for the BAC clone VVCS1H018A11 sequence and in Mb for the chromosome 7 of PN40024 genotype. The 11 genes deleted on chromosome 7 of the dwarfed plants are symbolized in blue.
